# Exploring the potential of blockchain technology for citizen engagement in smart governance

**DOI:** 10.12688/openreseurope.16153.1

**Published:** 2023-10-23

**Authors:** Niccolò Testi, Rebecca Marconi, Edna Pasher

**Affiliations:** 1 Università degli Studi di Macerata, Macerata, Marche, Italy; 2 Edna Pasher Phd and Associates Management Consultants Ltd, Tel Aviv, Israel

**Keywords:** smart governance, blockchain, smart city, citizen participation

## Abstract

This review article explores the potential of blockchain technology (BCT) as a key enabler for fostering transparency, trust, and citizen engagement in smart governance within smart cities. By examining the benefits of BCT in various aspects of smart city systems, such as data security and privacy, the paper highlights the advantages of implementing consortium blockchain architecture and the Proof of Authority (PoA) consensus algorithm and discusses the challenges of scalability and security concerns. Based on the literature showed in this article, we believe that the use of BCT in smart governance could significantly enhance citizen participation and help manage and deliver public services, paving the way for more effective decision-making processes and improved quality of life for citizens.

## Introduction

A smart city is an urban area that employs advanced technology to gather, analyse, and manage data to optimize various aspects of city life, with the final aim of improving citizens’ quality of life ^1^. Technology-based solutions in smart cities make use of technologies such as Information & Communication Technologies (ICT), Big Data, machine learning, automation, Artificial Intelligence (AI), the Internet of Things (IoT), and blockchain ^2^ to address economic, social, and environmental issues such as resource inefficiency, traffic congestion, air pollution, inadequate healthcare, lack of affordable housing, insufficient energy supply, and poor waste management ^3, 4^. In order to solve these issues, policymakers need to adopt a more participatory and bottom-up governance approach where citizens play an active role in policy-making processes, transforming the way smart cities are managed ^5^. The Organisation for Economic Co-operation and Development (OECD) identifies three key practices in the relationship between public institutions and citizens that promote a participatory process: information, consultation and active participation ^6^. From the point of view of the role of public institutions and therefore policymakers, information is a unidirectional relationship in which the institution produces and provides information useful for the social life of citizens (one-way relationships), consultation is a bidirectional relationship in which citizens provide information and data useful to public institutions (two-way relationships), and active participation is a collaborative process in which citizens are an integral part of the process of decision-making and policy-making and therefore both parties benefit from the relationship (advanced two-way relation). The new digital environment is fostering the development of a new system of public governance, offering new opportunities for citizen engagement and participation, and encouraging collaborative approaches to setting policy priorities and defining public services. In this respect, public governance is being called upon to change its approach, moving from a citizen-centred approach, where governments anticipate the needs of citizens and businesses, to a citizen-driven approach, where citizens and businesses can communicate their own needs ^7^ and address them in cooperation with public authorities ^8^. By promoting the exchange of knowledge, new ideas, methods and lessons learned, cities can encourage social cohesion and conversations. 9 define “conversing cities” as those that prioritize communication, networking, sharing ideas, and creating new knowledge.

This shift towards citizen involvement in urban planning and decision-making enables better alignment of public policies with community needs, fostering more inclusive, transparent, and democratic urban environments ^7^. As highlighted also in the Digital Compass ^10^, the European way for the digital society foresees the need for open democracy initiatives to contribute to inclusive policy-making as an enabler approach to improve participative governance and public support for democratic decisions. To this end, new technologies can play a crucial role not only in supporting administrative processes but also in shaping governance outcomes and therefore strategic planning of public policies is required to foster a broad transformation of the public sector to optimise the impact of digital governance ^8^. 11 notes that smart city agendas have often failed to be citizen-centric due to their top-down approach, which can be reversed by using technologies such as blockchain technology (BCT). As the inventor(s) of BCT, Satoshi Nakamoto ^12^, first explained, a blockchain is a distributed, peer-to-peer, immutable database that can be made accessible to some or all stakeholders interested in the data stored in it. BCT can contribute to smart cities by breaking down technological silos, empowering users, helping them regain control over their personal data, and enabling mechanisms to effectively audit providers and public administrations ^13^ in sectors like healthcare, transportation, logistics, supply chain management, and public administration ^14^. Considering that it has been described as a “trust machine” ^15, 16^, BCT can be particularly utilized in the domain of smart governance, which aims to ensure transparency, trust in public services, and citizen participation in decision-making processes ^17, 18^. 19 identify several opportunities associated with implementing smart governance in cities. One of the key opportunities is the potential for increased openness, mass collaboration, and participation. Smart governance can enable citizens to participate more actively in decision-making processes and provide feedback on government policies and services. This can lead to more effective governance models that better reflect the needs and preferences of citizens. By leveraging technology to improve decision-making and collaboration among different stakeholders, governments can create more effective and responsive governance models that better serve their citizens. For example, Amsterdam has implemented a smart city initiative that uses technology to improve communication and collaboration between citizens and city officials. The initiative includes a digital platform that allows citizens to report issues and suggest improvements, as well as an open data portal that provides access to information about the city ^20^. Other examples are the technologies for electronic voting (e-voting), which is seen as a viable alternative to traditional paper-based voting for citizen participation in decision-making ^21^. For example, the Estonian government provides citizens with multiple channels to be actively engaged ^22^ and participate in local, national, and European elections through the i-Voting system ^23^. This latter promotes cross-border digital governance and strengthens the country's democracy, as the vote is signed and encrypted with the voter’s certificates ensuring procedural safeguards such as transparency, verifiability, accountability, reliability, and security. Through the work of the Centre of Excellence for Internet Voting (SCCEIV), Estonia intends to advance online voting and invest more efforts in BTC for participatory governance. In terms of citizen participation, Estonia is ranked 3rd on the e-Participation Index ^24^.

Nevertheless, to enable smart governance, data generated from urban environments must be open and trustworthy while ensuring data security and privacy at different administrative and geographical levels ^3, 25^, but the collection, storage, processing, and analysis of heterogeneous data from residents face vulnerability issues ^26, 27^. As 28 and 3 explain, BCT can be applied to smart cities in various ways to solve these issues. For example, in smart healthcare, BCT can help to improve data sharing and interoperability among different healthcare providers while ensuring patient privacy. In smart transportation, it can enable secure and efficient transactions between different stakeholders such as passengers, drivers, and service providers. Using BCT in smart cities could increase transparency, security, efficiency, and accountability, and help to reduce fraud and corruption by providing a tamper-proof record of transactions. It can also enable more efficient use of resources by automating processes and reducing intermediaries. Additionally, BCT can enhance trust among different stakeholders by providing a decentralized platform for data sharing and collaboration. An example of this is the #SmartME project, presented by 29 and 30, which aims to transform Messina, Italy, into a smart city by creating an infrastructure and ecosystem of services using existing devices, sensors, and actuators. The project focuses on software development to tackle challenges like interoperability, networking, and security. It enables a decentralized, trust-less open data system for environmental sensing data acquisition, storage, and consumption. This real-world implementation demonstrates the success of a trust-less open data system allowing institutions to pool resources without relying on a third party. Thus, in the field of smart governance, BCT can be used to create a decentralized and secure platform for citizens to access public services and participate in decision-making processes. For example, Estonia is the first state to use BTC on a national level, as it started testing the technology in 2008 in response to the 2007 cyber-attacks and has been producing BTC for both the public and private sectors since 2012, becoming a pioneer in this field. The country uses the “Keyless Signature Infrastructure” (KSI) blockchain stack with the main objective of enforcing the integrity of government data and systems and e-services, and there are specific government registries supported by BTC, such as the health registry, property registry, business registry, inheritance registry, digital court system, monitoring and tracking information system, official government announcements, and the government gazette ^31^. In 2020, for the first time, the European Union assigned the eIDAS accreditation to the KSI BTC as a trust mark for qualified trust services with legal power for electronic transactions in the European Single Market ^32^.

According to the European Commission’s report on eGovernment Benchmark 2020, Malta and Estonia are the European frontrunners in eGovernment, followed by Austria and Latvia, while Luxembourg, Hungary and Slovenia show the most significant progress in recent years ^33^.

Despite the advantages of BCT for smart governance, some technical challenges remain as individuated by 34. Also, it must be reminded that a challenge for smart governance is the potential for increased inequality if not all citizens have access to technology ^19^. Smart governance relies on the use of technology to improve decision-making and collaboration, but not all citizens may have access to this technology or be able to use it effectively. This could lead to a situation where some citizens are left behind or excluded from important decision-making processes.

This review article explores the potential of BCT as a key enabler for fostering transparency, trust, and citizen engagement in smart governance within smart cities, while also individuating some challenges. The paper is structured as follows. The next section presents the method followed for the literature review conducted for this exploratory study. Then, an overview of BCT-enabled smart governance based on the literature review is presented. The overview first introduces the potential of BCT to enhance transparency, trust, and collaboration in smart governance, focuses on BCT-enabled e-voting for citizen participation, and highlights the role of BCT in ensuring data security and privacy in smart city systems and infrastructure. Also, it discusses the challenges and limitations of implementing BCT in smart city ecosystems and presents the advantages of using consortium blockchains and the Proof of Authority (POA) consensus algorithm for implementing blockchain-enabled smart governance. A discussion of the literature is presented. Finally, conclusions from the literature are drawn, implications for policymakers are provided, and avenues for future research are suggested.

## Methods

In this exploratory study, a qualitative research approach was utilized to investigate the potential of BCT for citizen engagement in smart governance through a literature review. Given the exploratory nature of the study, no strict selection criteria were employed, and the literature review was conducted inclusively rather than exclusively, with the aim of casting a wide net over the existing body of knowledge. Databases for scientific literature, such as Scopus, ScienceDirect, Web of Science were searched in February 2023. The search was not limited to articles directly focusing on the application of BCT in smart governance, but also included related research that discussed either aspect individually or other tangentially related topics. The key phrases used for the search included, but were not limited to, "blockchain technology", "citizen engagement", "citizen participation", "smart governance", "e-governance", and "e-voting". Each selected article was examined to extract valuable insights relevant to the research objective. Aspects such as the potential advantages, challenges, case studies, practical examples, and theoretical perspectives on the use of BCT in engaging citizens within the context of smart governance were addressed. Given the qualitative nature of the research, the data was synthesized in a narrative form, with the key findings and thematic patterns identified and discussed ^35^.

## BCT-enabled smart governance

### BCT for citizen participation in decision-making processes

Researchers addressed the potential of BCT in creating more inclusive, democratic, and transparent urban governance models by empowering citizens to participate actively in decision-making processes and policy implementation. Researchers proposed diverse blockchain-based systems to enable citizens to actively partake in decision-making processes, evaluate shared information quality, and contribute to policy formulation and implementation. 11 suggests a blockchain-based smart contract platform where citizens share needs and opportunities to be addressed at the community level. This platform is provided by the local administration, and the collaborative results are endorsed by the city council. In this system, citizens decide on the actions to be taken by the community and what actions the city council should take to address the unmet needs. 7 propose a public participation consortium blockchain system for infrastructure maintenance. This system allows citizens to actively participate in the decision-making process and observe administrative procedures in real-time. The blockchain architecture proposed enables the involvement of a verifier group, randomly and dynamically selected from public citizens, to participate in transaction verification. 36 propose a blockchain framework focusing on social, environmental, and economic aspects, as well as established policies and good practices, to offer solutions to stakeholders. It dynamically identifies and proposes new policies, regulations, and initiatives based on social trends and citizen maturity levels. The framework aims to boost citizen participation and inclusion in governance models by offering secure, robust, and flexible solutions that enable interconnected citizens, smart devices, big data analytics, and cloud computing. BCT facilitates citizen participation by ensuring secure and transparent transactions resistant to tampering. Citizens can utilize blockchain-based applications for digital IDs, voting systems, financial applications, and contracts. 37 investigate the potential of BCT in incentivizing democratic participation and citizen engagement in smart cities. Smart contracts as policies are executed based on the data-driven choices of the community. The user actively participates through data-informed votes on policies, influencing behaviours on urban water resource management policy negotiation. 38 present a blockchain-based reputation system aimed at improving decision-making in smart cities by enabling citizens to evaluate the quality of shared information. Participants can validate or dispute the information, with individual reputations influencing credibility. Reputation acts as a kind of currency, with citizens betting on their reputation when offering opinions and regaining reputation if proven correct. Municipal stakeholders oversee the system, making final judgments on information quality and adjusting associated reputations accordingly. BCT is employed to decentralize stakeholders' control over reputation management, ensuring the reliable dissemination of information, such as accident reports.

All of these proposed systems emphasize the importance of transparency, decentralization, and secure transactions in fostering greater citizen involvement. The use of BCT in these systems ensures that transactions are tamper-resistant and allows for the real-time monitoring of administrative procedures. The integration of smart contracts, digital IDs, voting systems, and other blockchain-based applications promotes an interconnected ecosystem involving citizens, smart devices, big data analytics, and cloud computing.

### BCT-enabled e-voting for citizen participation

39 examine the potential advantages and disadvantages of e-voting, particularly focusing on the security, accessibility, and accuracy of various e-voting technologies. Potential benefits of e-voting are increased voter participation, improved efficiency, and reduced costs. However, the authors also emphasize the importance of addressing the challenges associated with e-voting, including voter privacy, system security, and the potential for fraud or manipulation. 40 conducted a systematic review of the challenges and opportunities of applying BCT for e-voting. They found that BCT offers several benefits for e-voting, such as reduced long-term costs compared to traditional secure data storage systems, instant and secure election results, and increased confidence in the voting process, potentially leading to higher voter participation. However, there are challenges, including scalability attacks, reduced transparency, the use of untrusted systems, resistance to coercion, and unknown security risks that require further testing. Additionally, blockchain-based e-voting systems may necessitate more complex software designs and advanced management skills. Despite these challenges, it was found that BCT can enhance the security and transparency of elections by preventing data manipulation, maintaining data integrity, creating a safer environment through permissioned blockchain structures and independent control nodes, and facilitating secure and transparent voting processes through interconnected nodes. Furthermore, blockchain-based voting systems can address common e-voting concerns by improving privacy protection and transaction speed. BCT could enable secure and transparent e-voting due to its properties of transparency, decentralization, irreversibility, and nonrepudiation, without the need for a trusted third party ^21^. Blockchain-based voting platforms can be used to create a decentralized, secure, and unique voting system that eliminates the complexities and inefficiencies of traditional voting methods. These platforms enable citizens to provide feedback and raise grievances, which can be considered and resolved, ultimately improving smart city facilities ^41^. 17 propose a system architecture for efficient citizen data collection, processing, storage, and permissioned sharing of data across geographical administrative boundaries in smart cities. Districts within a city have permissioned blockchains that register votes from the citizens, which are processed with edge computing and then sent to the city administration's shared blockchain visible to all. At the same time, to incentivize stakeholders’ participation, 41 suggest using BCT to develop a blockchain-based loyalty and rewards platform, ensuring that the right contributors from society are rewarded for their contributions to smart city development. 42 also emphasize the value of "Virtual Tokens" to reward citizen participation and promote co-management of public utility projects. Combining these ideas, a comprehensive blockchain-based platform could effectively encourage citizen engagement and contribution to the development of smart cities.

However, the problems of voting, also affecting e-voting, such as free-riding and tyranny of the majority, can have significant implications for the fairness and representativeness of democratic decision-making processes. Free riding occurs when individuals choose not to participate in collective decision-making, relying instead on the efforts and opinions of others. This phenomenon can arise from various factors, including a lack of information, voter apathy, or the belief that one's vote will not make a significant difference, and can be problematic as it leads to lower voter turnout and potentially skewed election results ^43^. Consequently, the decisions made through e-voting may not accurately represent the preferences of the entire electorate. Indeed, a study by 44 explored the impact of e-voting on political participation, with a focus on Estonia's 2007 parliamentary elections, the first to offer both traditional polling and internet voting options. The author found that e-voting primarily substituted for traditional voting rather than attracting new voters. Moreover, the politically well-established groups, rather than underrepresented social groups, predominantly engaged in e-voting, and the new voting technology had a non-neutral political effect, with e-voters favouring parties supported by the ethnic majority and wealthier areas. Consequently, the author concluded that e-voting may exacerbate political participation inequalities rather than mitigate them. Another problem not specifically regarding e-voting but voting in general is the tyranny of the majority, which refers to a situation where the majority imposes its will on the minority, disregarding their rights and interests. The tyranny of the majority can manifest when the majority's preferences consistently override minority viewpoints, leading to a lack of diversity and inclusiveness in decision-making ^45^. This problem can be exacerbated by factors such as polarized opinions, groupthink, and the absence of mechanisms to protect minority rights ^46^. As a result, decisions made through e-voting may not be equitable or fair to all members of society.

### BCT for data security and privacy in smart cities

Data security and privacy are crucial aspects of smart city systems and, consequently, smart governance. As for data security, BCT has been proposed as a solution to these security challenges due to its decentralization, transparency, reliability, security, and immutability ^28, 47^. Integrating BCT can help eliminate many of the issues faced by IoT, supporting smart cities ^28^. 26 describe a new procedure for designing and implementing a decentralized platform that combines IoT and BCT with smart transportation systems. 48 have designed and implemented a decentralized, trust-less DLT-based system for environmental sensing, data acquisition, storage, and consumption in the context of a real-world smart city deployment. The system is user-friendly and allows for trust-less data audit, requiring IoT nodes to authenticate themselves when sending readings. 49 suggest a solution to data security and integrity problems that utilizes Hyperledger Fabric and Inter-Planetary File System (IPFS), taking advantage of blockchain's immutability and decentralization to boost transparency and integrity while guaranteeing data availability. This innovative method enables users to independently confirm data integrity and retrieve data logs, enhancing trust and minimizing the likelihood of making wrong decisions based on inaccurate data. As for data privacy, the challenge for smart city designers and planners is to ensure the required levels of trust in security and privacy so that citizens can trust sharing and using data ^27^. 13 suggests that actors interacting with smart city systems can use blockchain wallet-type applications to own and control their data, and at the same time, the blockchain can be used to verify that the data is authentic and up to date. Additionally, 50 state that blockchain-based identity management can provide a secure and trustworthy environment for accessing services within a smart city, enabling seamless interaction with various city services and leading to enhanced user experiences and increased trust in the smart city infrastructure.

### Challenges for the integration of BCT in smart cities

As seen in the previous paragraphs, BCT can enable more transparency and trust for including citizens in smart governance decision processes, however, some challenges to the integration of BCT in smart cities are to be overcome. As a relatively new technology, blockchain is still in its early development stages, and aspects such as performance, security, and scalability remain uncertain ^51^. Moreover, both blockchain and smart cities are in their infancy and require significant research efforts for integration ^2^. 34 identify challenges related to the integration of BCT in smart city ecosystems. One of the primary challenges, also mentioned by 52, is scalability, as data sets in smart cities can be of enormous sizes. 34 and 53 propose using IPFS decentralized cloud storage in smart cities for off-chain storage of data, while only the data’s hashes are uploaded to the blockchain for reference. Another critical challenge, according to 34, is maintaining unique identification for all participating nodes. Since nodes are identified by unique sets of public-private keys, and the security of these keys is of vital importance, a compromise by a cybercriminal or a careless holder can have severe consequences. 51 also mention the rigidity of blockchain governance: once the rules of governance are established in a blockchain or smart contracts that are almost or completely immutable by design, it becomes challenging or impossible to modify them, which contradicts the need for adaptability and corrigibility of smart governance.

### Consortium blockchain architecture and POA consensus algorithm for smart governance

We argue that, when deciding to implement BCT in smart governance, policymakers would need to define the technical aspects of the blockchain solution they want to adopt, because these influence governance. As 54 explained, Blockchain governance can be permissionless or permissioned, depending on its ownership and rules . Governance in permissionless blockchains is fully decentralized and allow any user in the network to read, write, or audit the information stored in them. All users are anonymous, ensuring the privacy of their personal information. Since anonymity in transactions could increase moral hazard and, consequently, perceived risk, the transactions are recorded, made immutable, and visible to everyone. This creates a trust-less environment where trust among nodes is not necessary. On the contrary, permissioned blockchains can be owned either by one entity (private blockchain) or multiple equally powered actors (consortium blockchain), who have full control of the blockchain and can set different levels of accessibility and writing and reading rights to users. Contrarily to permissionless blockchains, in this case, all users are known but the transactions can be kept private. Since all actors are known and accountable for their actions, they might be incentivized to act ethically ^55^, contrary to what happens in permissionless blockchains where every actor is protected by anonymity. However, even full accountability does not eliminate the risk of fraudulent behaviour ^56^. Anyways, permissioned blockchains give total access control to their owner(s), hence, should some actors begin to act maliciously, they can be quickly removed from the network. Moreover, permissioned blockchains restrict access to data ^57^ which may be sensitive ^58^. Finally, permissionless blockchains are often found impractical due to their slowness in verifying transactions, high costs, and lack of confidentiality of the information uploaded to them ^59^. Instead, permissioned blockchains perform better in terms of transaction throughput speed ^60^. The average time of a transaction being validated can be of milliseconds and this could even enable real-time auditability of information as soon as it is uploaded to the blockchain ^61^. While being different in terms of the level of decentralization, both private and consortium blockchains share the advantages of a faster transaction throughput speed, compared to permissionless blockchains, and the possibility for the owners to amend the information already uploaded by appending a new version of it 60. While being considered a good feature of blockchains, the immutability of information might not always be desirable. For example, changing the data uploaded to a BC could be necessary if they contain errors. In permissioned blockchains, the change can be appended to the chain of blocks, and a new version of the original information is created. The original data is not removed and can still be accessed. Also, the blockchain keeps track of these operations and who did them, making the process transparent ^60, 62^. To verify that the data have not been modified with malicious intent, a hybrid blockchain architecture can be used, where the permissioned blockchain stores sensitive data which is then notarized on public blockchains, making the data’s hashes immutable and retrievable to check the immutability of the data on the permissioned blockchain ^63^.

For the reasons mentioned before, permissioned blockchains could be more suited for smart city governance, compared to the permissionless ones. While the permissionless (public) type allows for more transparency and is truly distributed, it also suffers from a lack of control on the blockchain and scalability, compared to permissioned BCs (private and consortium). Consortium blockchains might provide a better solution when compared both to permissionless and private blockchains because they mitigate some of the risks of private blockchains by removing centralized control ^60^. A private blockchain is owned by a single entity that has total top-down control over the write and read rights and the validation process. The structure of a private blockchain might look decentralized because the data contained in it are usually distributed among multiple nodes. However, these are controlled by the owner of the blockchain or by other parties that the owner pre-approved, so private blockchains are centralized. The owner of a private BC can unilaterally choose to restrict access to some information, or to avoid writing certain transactions, modify or remove them altogether, even if performing these actions would lead to reputational damage for the owner, if caught ^64^. For these reasons, when a private blockchain is used, stakeholders must have a high level of trust in the owner of the blockchain. In the hypothetical scenario in which a municipality used a private blockchain to write data from all the smart city’s stakeholders, then trust would not be created, and a consortium blockchain should be used. Consortium blockchains are more decentralized since control over the BC is shared among multiple owners, instead of being centralized in the hands of a single entity. In consortium BCs, some equally powered nodes of the network administer the blockchain and have special rights and functions: deciding who can become a node of the network and who must be kicked out for violating the rules; granting writing and reading rights to other nodes; validating transactions, mining and appending blocks ^65^. These administrators, sometimes called validators, are often pre-determined at the genesis of the BC and are usually its original owners. In some consortium blockchains, validators can vote to add or expel other validators ^64^. As 7 explain, the reasons for employing a consortium blockchain in smart city governance are twofold. First, as the population and the number of infrastructures continue to grow, the blockchain for infrastructure maintenance must be efficient and scalable, which hinders the application of public blockchains. Second, multiple organizations will be involved in collaboratively implementing the infrastructure maintenance process, rendering private blockchains unsuitable. Therefore, consortium blockchains provide an ideal balance between the need for scalability, efficiency, and collaboration among various stakeholders in the smart city context, which is an opinion shared by 29 too.

Another technical aspect to be considered is the kind of consensus algorithm to use in blockchain-enabled smart governance. Many consensus algorithms exist, each one with its characteristics ^66^. The consensus algorithm plays a vital role in BCT, particularly in ensuring public participation and maintaining the system's security and efficiency. Given BCT’s low scalability limit, 7 highlight the importance of real-time interaction and the complexity of involving public users, calling for a low-latency and cost-efficient consensus protocol. 11 proposes the use of the Proof of Authority (PoA) algorithm as a solution to these challenges. PoA is based on identity and reputation, which reduces the need for mining and expensive computational operations, leading to higher transaction throughput. With PoA, participants become validators and earn mining rewards, incentivizing them to maintain their reputable position. PoA network operators can be city councils or national governments, allowing citizens to participate through mobile apps and wallets. Nodes are added to the network after permission is granted by the network operator, and oracles can be called for external data inputs when necessary.

## Discussion

In this paper, we postulate that BCT could enable more inclusive, democratic, and transparent smart governance in smart cities. Smart cities systems can use BCT-enabled data decentralization, transparency, reliability, security, and immutability ^28, 47^ to increase data security and privacy and foster trust among citizens ^50^. Researchers have proposed various blockchain-based systems that empower citizens to actively participate in decision-making processes ^7, 11^, evaluate shared information quality ^38^, and contribute to policy formulation ^36^ and implementation ^37^. Another application field of BCT is that of e-voting, which offers the potential to improve citizen participation by enhancing security, accessibility, and accuracy ^39^. The benefits of e-voting include increased voter participation, improved efficiency, and reduced costs ^40^. BCT-based voting systems can also address common e-voting concerns by improving privacy protection and transaction speed. However, e-voting may encounter problems such as free riding, which can lead to lower voter turnout and skewed election results ^43^, and the tyranny of the majority, which can result in a lack of diversity and inclusiveness in decision-making ^45^. In our opinion, these issues highlight the need to carefully consider the implementation of BCT-enabled e-voting to ensure equitable and fair representation for all members of society.

Despite the potential of BCT for smart governance, we also found challenges that need to be addressed when integrating BCT into smart cities. These challenges include performance, security, and scalability ^51^. Smart cities can generate vast amounts of data, presenting challenges for blockchain scalability ^52^. Solutions such as IPFS decentralized cloud storage have been proposed for off-chain data storage, with only the data's hashes uploaded to the blockchain for reference ^34, 53^. Regarding security, maintaining unique identification for all participating nodes is crucial, as nodes are identified by unique sets of public-private keys and a compromise by a cybercriminal or careless holder can result in serious security issues ^34^. Finally, blockchain governance may be too rigid. Once established, the rules of governance in a blockchain or smart contracts are often immutable by design, making them difficult or impossible to modify. This rigidity contradicts the adaptability and corrigibility requirements of smart governance ^34^. We argue that addressing these challenges is essential for realizing the full potential of BCT in smart cities and fostering more inclusive and transparent governance systems.

Considering the technical aspects of implementing BCT in smart governance, consortium blockchains appear to be a more suitable choice for smart cities because they offer a balance between decentralization and control, ensuring efficiency, scalability, and collaboration among various stakeholders, and allow for faster transaction throughput and the possibility to amend information while maintaining transparency and accountability ^7, 29^. We suggest that a combination of consortium blockchain architecture and the Proof of Authority (PoA) consensus algorithm based on identity and reputation ^11^ would to be particularly well-suited for smart governance applications.

In conclusion, we believe that the adoption of BCT in smart governance has the potential to revolutionize the way local governments manage and deliver services in smart cities allowing for improved collaboration, efficiency, and transparency in decision-making processes. To realize these benefits, some challenges related to the implementation of this technology need to be addressed, such as low scalability and potential security issues due to poor public-private keys management. We argue that policymakers should carefully consider the technical aspects of implementing BCT, such as the choice of blockchain architecture and consensus algorithm and address the challenges of BCT integration in smart cities. Specifically, we suggest that the adoption of consortium blockchain architecture and the PoA consensus algorithm can provide an effective foundation for implementing smart governance in smart cities, due to its balance between decentralization and control, higher transaction throughput, and scalability.

Several avenues for future research can be suggested since this paper was an explorative study. First, in-depth research on different blockchain types and consensus algorithms should be conducted to identify the most suitable solutions for diverse smart governance scenarios. Second, researchers could investigate the potential synergies between BCT and other emerging technologies like AI, IoT, and edge computing, to enhance smart city governance and create more efficient and sustainable urban ecosystems. Third, there is a need for studies on the potential benefits and drawbacks of implementing BCT in smart governance, such as the impact on citizen engagement, job creation, and the overall quality of life in smart cities. Also, more research is needed on what are the necessary skills and knowledge required by policymakers, city administrators, and other stakeholders to effectively implement and manage blockchain-enabled smart governance systems. Finally, since research on BCT for smart governance has been mainly theoretical by now, there is a need for pilot projects and real-world case studies of blockchain-based smart governance implementations to gather insights, assess effectiveness, and identify best practices for large-scale deployment.

## Ethics and consent

Ethical approval and consent were not required.

## Data Availability

No data are associated with this article.
