# Supplementary material for: Exploring the potential of blockchain technology for citizen engagement in smart governance
Source: Open Res Eur. 2025 Jan 3;3:183. Originally published 2023 Oct 23. [Version 4] doi: 10.12688/openreseurope.16153.4 (PMC11809474; doi:10.12688/openreseurope.16153.4)
Supplement: Peer review history

# Reviewer report: Reviewer response for version 4

**DOI:** 10.21956/openreseurope.20799.r49464

**Published:** 2025-01-06

**Authors:** .J. Ebenesar Anna Bagyam

**Affiliations:** 1 Karpagam Academy of Higher Education, Coimbatore, Tamil Nadu, India

The corrections align well with the reviewer’s recommendations and significantly enhance the article's depth and balance.

Are all factual statements correct and adequately supported by citations?

Partly

Is the review written in accessible language?

Partly

Are the conclusions drawn appropriate in the context of the current research literature?

Yes

Is the topic of the review discussed comprehensively in the context of the current literature?

Partly

Reviewer Expertise:

Information Technology and Smart Systems, Operations Research, Queueing Systems

I confirm that I have read this submission and believe that I have an appropriate level of expertise to confirm that it is of an acceptable scientific standard.

# Reviewer report: Reviewer response for version 3

**DOI:** 10.21956/openreseurope.18671.r47311

**Published:** 2024-12-26

**Authors:** Gasim Othman Alandjani

**Affiliations:** 1 Yanbu Industrial College, Yanbu, Saudi Arabia

Review Report

**Summary of the Article**:

 The manuscript explores the role of blockchain technology (BCT) in enhancing citizen engagement and governance in smart cities. It reviews current literature, presents case studies, and discusses the potential benefits and challenges associated with implementing BCT in urban governance.

**Comprehensiveness in Literature Context**:

**Partly**

 While the article covers important aspects of BCT in governance, it lacks depth in discussing recent developments and critiques in the literature. The authors should include more recent studies and counterarguments to provide a balanced view.

**Factual Accuracy and Citations**:

**Partly**

 Most factual statements are supported; however, some claims need clearer citations. For instance, specific examples of successful BCT implementations should be substantiated with recent data or case studies.

**Accessibility of Language**:

**Partly**

 The language is generally accessible; however, certain technical terms and concepts are not adequately explained to readers without a background in blockchain technology. A glossary or simplified explanations would enhance understanding.

**Conclusions Appropriateness**:

**Partly**

 The conclusions drawn are relevant but could be strengthened by summarizing the main findings more clearly and offering specific recommendations for future research.

**Constructive Suggestions**:

1. **Expand Literature Review**: Include recent studies and critiques to provide a more comprehensive view of the current state of research.
2. **Clarify Technical Terms**: Define complex terms and concepts to improve accessibility for a broader audience.
3. **Support Claims with Data**: Provide specific examples and data to substantiate claims about BCT’s effectiveness in governance.
4. **Strengthen Conclusions**: Summarize key findings and offer clear recommendations for policymakers and future research directions.

Addressing these points will enhance the manuscript's scientific soundness and overall impact.

Are all factual statements correct and adequately supported by citations?

Partly

Is the review written in accessible language?

Partly

Are the conclusions drawn appropriate in the context of the current research literature?

Yes

Is the topic of the review discussed comprehensively in the context of the current literature?

Partly

Reviewer Expertise:

Computer Science and Engineering, Blockchain, AI, IoT, Deep Learning, Network Security

I confirm that I have read this submission and believe that I have an appropriate level of expertise to confirm that it is of an acceptable scientific standard, however I have significant reservations, as outlined above.

# Reviewer report: Reviewer response for version 3

**DOI:** 10.21956/openreseurope.18671.r46578

**Published:** 2024-12-18

**Authors:** .J. Ebenesar Anna Bagyam

**Affiliations:** 1 Karpagam Academy of Higher Education, Coimbatore, Tamil Nadu, India

**Summary of the Article**

 This paper reviews blockchain technology and its applications in smart cities to explore its benefits in sectors like governance, transportation, healthcare, and energy management in improving security, transparency, and efficiency. It discusses its potential, challenges, and future directions but lacks depth in certain areas such as technical details, real-world implementation challenges, and critical research gaps.

**Review Report:**

**Comprehensiveness in Current Literature**

**Answer: Partly**

 The article covers the topic generally but is not very detailed about particular blockchain platforms, technical challenges such as scalability and interoperability, and practical applications. It should provide more in-depth case studies and discussion on the gaps in research.

 Recommendations: Add more technical discussion and real-world examples; also, identify particular gaps in research.

**Factual Correctness and References**

**Answer: Partly**

 Most claims are valid, but parts about scalability and regulatory challenges need more citations. Claims regarding blockchain's advantages may have been supported by more examples and studies.

 Recommendations: Parts to be strengthened by additional citations especially on the scalability and regulatory issues.

**Language Access**

**Answer: Yes**

 The article is written in plain language and can easily be read by any audience even without technical backgrounds.

 Recommendations: No suggestions for changes.

**Conclusion and Literature Background**

**Answer: Partially**

 The conclusions are in line with the literature but need to reflect blockchain's scalability, regulatory issues, and social acceptance.

 Recommendations: The conclusions should be revised to reflect the challenges and limitations more explicitly.

**Final Recommendations**

 Technical depth on blockchain platforms and case studies should be added.

 Increase citations to support claims, especially on challenges.

 The conclusions should be revised to reflect the complexities of blockchain integration and address the scalability and regulatory concerns.

Are all factual statements correct and adequately supported by citations?

Partly

Is the review written in accessible language?

Partly

Are the conclusions drawn appropriate in the context of the current research literature?

Yes

Is the topic of the review discussed comprehensively in the context of the current literature?

Partly

Reviewer Expertise:

Information Technology and Smart Systems, Operations Research, Queueing Systems

I confirm that I have read this submission and believe that I have an appropriate level of expertise to confirm that it is of an acceptable scientific standard, however I have significant reservations, as outlined above.

# Reviewer report: Reviewer response for version 3

**DOI:** 10.21956/openreseurope.18671.r46587

**Published:** 2024-12-13

**Authors:** Negar Noori

**Affiliations:** 1 Rotterdam School of Management, Erasmus university of Rotterdam, Rotterdam, The Netherlands

At the beginning of the introduction, after defining the concept of a smart city and highlighting its various applications, it is mentioned that to address and solve economic, social, and environmental issues, policymakers need to adopt a participatory decision-making approach. However, it is not clearly explained how this approach can help policymakers find better solutions or why it is important to shift towards a bottom-up governance approach in smart cities.

 At the end of the first paragraph, a new city label (or brand) is introduced as the "conversing city," but it is somewhat confusing how this relates to the smart city concept. Are we talking about smart cities or conversing cities here? I also disagree with the claim that all smart city agendas have a top-down approach and often fail because of it. This is a strong opinion that requires robust supportive arguments or references. Additionally, regarding the second part of the sentence, while BCT can be a technological solution, it cannot serve as a policy tool to change the top-down approach.

 Regarding the methodology, although the authors mention that there are no selection criteria or inclusion/exclusion parameters, it would be helpful to provide information about the number of papers or studies reviewed and included. This would offer more insight into the breadth and scope of the study.

 Both the review and discussion sections remain overly descriptive. Tabulating the key findings, including key themes, sub-themes, and associated attributes based on the thematic analysis conducted, would be a valuable option to consider.

Are all factual statements correct and adequately supported by citations?

Partly

Is the review written in accessible language?

Yes

Are the conclusions drawn appropriate in the context of the current research literature?

Partly

Is the topic of the review discussed comprehensively in the context of the current literature?

Yes

Reviewer Expertise:

Smart Cities, Digital governance, digital inclusion and digital transformation policy

I confirm that I have read this submission and believe that I have an appropriate level of expertise to confirm that it is of an acceptable scientific standard, however I have significant reservations, as outlined above.

## References

[1] : A Smart Governance diffusion model for blockchain as an anti-corruption tool in Smart Cities. *Journal of Smart Cities and Society* .2022;1(1) : 10.3233/SCS-210122 71-92 10.3233/SCS-210122

# Reviewer report: Reviewer response for version 2

**DOI:** 10.21956/openreseurope.18276.r36611

**Published:** 2024-01-15

**Authors:** Evrim Tan

**Affiliations:** 1 Public Governance Institute, Katholieke Universiteit, Leuven, Belgium

Thank you for your revisions. I only have some minor comments regarding your amendments: 1) You wrote: "the integration of the Decidim ^26^ digital platform for citizen participation in Barcelona with DLT for citizen-led co-production". However, during the research period, a complete integration did not occur. The study was based on a pilot that showcased how DLT technology could be utilized with the Decidim platform for citizen petitions. I am not aware that a full integration has taken place yet. Therefore, I suggest you correct that sentence. 2)  You wrote: "For example, if a small group of influential individuals dominate off-chain governance processes, what appears to be a centralized governance structure might function similarly to a semi-centralized or polycentric system. Conversely, in PoW and PoS-based systems, if on-chain governance is controlled by a few major operators with significant control over mining resources or token holdings, a system initially designed to be decentralized could operate more like a semi-centralized or polycentric governance structure." I believe in the first sentence, 'centralized' should be 'decentralized', otherwise the sentence does not make much sense. Also please cite the work that mentions this possible shift (ref. number 64).

Are all factual statements correct and adequately supported by citations?

Partly

Is the review written in accessible language?

Yes

Are the conclusions drawn appropriate in the context of the current research literature?

Yes

Is the topic of the review discussed comprehensively in the context of the current literature?

Partly

Reviewer Expertise:

NA

I confirm that I have read this submission and believe that I have an appropriate level of expertise to confirm that it is of an acceptable scientific standard.

# Reviewer report: Reviewer response for version 1

**DOI:** 10.21956/openreseurope.17437.r35924

**Published:** 2023-11-16

**Authors:** Evrim Tan

**Affiliations:** 1 Public Governance Institute, Katholieke Universiteit, Leuven, Belgium

This is a well-constructed and argued article, and I commend the works of the authors. I've noted some minor suggestions to further enhance their work:

 - Decidim is also a widely used digital platform in Spain and Italy for e-voting and citizen participation. Additionally, Tan & Rodriguez (2023) have delved into different forms of citizen-led co-production using Decidim with DLT in the case of Barcelona. The authors could reference this work to illustrate a practical application of blockchain in citizen participation scenarios.

 - Permissionless systems, while decentralized, may evolve into semi-centralized or poly-centric networks over time, as evidenced by even the most decentralized networks like Bitcoin (Tan et al, 2022). Including this information would be beneficial.

 - The potential for change in permissionless blockchains exists but is more complex, often necessitating forking and the majority consensus of network participants.

 - The authors propose PoA as a potential solution to address certain limitations of widely used consensus mechanisms (e.g., PoW, PoS) but do not provide an explanation of these alternative consensus mechanisms. Furthermore, the authors have omitted a citation to the significant PoA system in Europe, the European Blockchain Services Infrastructure (EBSI). I recommend that the authors provide more context about consensus mechanisms and EBSI. For insights into the implementation of EBSI for specific use cases, the authors can refer to Tan et al (2023).

Are all factual statements correct and adequately supported by citations?

Partly

Is the review written in accessible language?

Yes

Are the conclusions drawn appropriate in the context of the current research literature?

Yes

Is the topic of the review discussed comprehensively in the context of the current literature?

Partly

Reviewer Expertise:

blockchain in public governance

I confirm that I have read this submission and believe that I have an appropriate level of expertise to confirm that it is of an acceptable scientific standard.

## References

[1] : Paths to citizens-controlled coproduction: The use of blockchain technology in digital coproduction. *Public Management Review* .2023; 10.1080/14719037.2023.2218388 1-20 10.1080/14719037.2023.2218388

[2] : Blockchain governance in the public sector: A conceptual framework for public management. *Government Information Quarterly* .2022;39(1) : 10.1016/j.giq.2021.101625 10.1016/j.giq.2021.101625

[3] : Verification of Education Credentials on European Blockchain Services Infrastructure (EBSI): Action Research in a Cross-Border Use Case between Belgium and Italy. *Big Data and Cognitive Computing* .2023;7(2) : 10.3390/bdcc7020079 10.3390/bdcc7020079
